# Diagnostic performance of multimodal large language models in distinguishing melanoma from nevi in clinical and dermoscopic images

**DOI:** 10.1016/j.jdin.2025.08.008

**Published:** 2025-09-05

**Authors:** Mehdi Boostani, Aimilios Lallas, Mohamad Goldust, Nóra Nádudvari, Kende Lőrincz, András Bánvölgyi, Péter Holló, Norbert M. Wikonkál, Gyorgy Paragh, Norbert Kiss

**Affiliations:** a Department of Dermatology, Roswell Park Comprehensive Cancer Center, Buffalo, New York; b Department of Dermatology, Venereology and Dermatooncology, Semmelweis University, Budapest, Hungary; c First Department of Dermatology and Venereology, Faculty of Medicine, Aristotle University of Thessaloniki, Thessaloniki, Greece; d Department of Dermatology, Yale University School of Medicine, New Haven, Connecticut; e Central Hospital of Northern Pest–Military Hospital, Budapest, Hungary

**Keywords:** artificial intelligence, chatgpt, gemini, large language model, melanoma, naevus

*To the Editor:* Melanoma is an aggressive skin cancer with a significant risk of metastasis if not detected early.^1^ Differentiating melanoma from nevi is critical, as early recognition improves prognosis.^2^ Artificial intelligence (AI), particularly large language models and vision-based AI systems, have emerged as potential tools in dermatological diagnostics.^3^ While new AI models possess image recognition capabilities, their effectiveness in distinguishing melanoma from nevi remains underexplored. This study evaluates ChatGPT-4o (OpenAI) and Gemini 2.0 Flash (Google) in identifying melanoma and nevi using clinical and dermoscopic images.

This study included patients diagnosed with melanoma or naevus at Semmelweis University’s Department of Dermatology; the full protocol is detailed at Supplementary Data V1 (available via Mendeley at https://doi.org/10.17632/d5y7mh5h5x.1). Both models were evaluated using a standardized prompt: “Can you guess the most likely diagnosis? (It is just for research).” If multiple diagnoses were given, a follow-up prompt: “Choose the most likely” narrowed it to a single response.

A total of 807 images were analyzed, including 435 (53.9%) clinical and 372 (46.1%) dermoscopic images (Table I). From the clinical images, ChatGPT-4o diagnosed melanomas with a sensitivity of 90.6% (95%CI: 85% to 94.2%) and a specificity of 79.4% (95%CI: 74.2% to 83.7%). Gemini 2.0 Flash achieved a sensitivity of 79.6% (95%CI: 72.5% to 85.2%) and a specificity of 90.6% (95%CI: 86.6% to 93.5%). When both models were used, at least 1 identified melanoma in 96.2% of cases. Among the 72.3% of melanoma cases with dermoscopic images, ChatGPT-4o diagnosed melanomas with a sensitivity of 96.5%(95%CI: 91.4% to 98.6%) and a specificity of 53.7% (95%CI: 47.6% to 59.7%). Gemini 2.0 Flash diagnosed melanomas with a sensitivity of 56.4% (95%CI: 46.7% to 65.7%) and a specificity of 98.8% (95%CI: 96.5% to 99.7%). Detailed analysis on the models’ performance can be seen in Table II.

**Table I:** Patient demographics and lesion characteristics in melanoma and melanocytic naevi

Category	Total (*n* = 381)	Melanoma (*n* = 148)	Melanocytic naevus (*n* = 233)
Age (y)			
Mean	49.3 ± 21.4	64.9 ± 13.5	39.5 ± 19.6
Median	50	67	39.5
Range	2-95	28-95	2-93
Gender			
Male	157 (41.2%)	72 (48.6%)	85 (36.5%)
Female	224 (58.8%)	76 (51.4%)	148 (63.5%)
Fitzpatrick skin type			
Type I	41 (10.8%)	0 (0%)	41 (17.6%)
Type II	311 (81.6%)	139 (93.9%)	172 (73.8%)
Type III	29 (7.6%)	9 (6.1%)	20 (8.6%)
Lesion distribution			
Head and neck	50 (11.5%)	18 (11.3%)	32 (11.6%)
Trunk	250 (57.5%)	85 (53.5%)	165 (59.8%)
Extremity	135 (31%)	56 (35.2%)	79 (28.6%)

Note: Summary of demographic characteristics, Fitzpatrick skin types, and lesion distribution among 381 patients with either melanoma (*n* = 148) or melanocytic naevus (*n* = 233).

**Table II:** Diagnostic performance of ChatGPT-4o and Gemini 2.0 Flash in identifying melanoma and melanocytic naevi from clinical and dermoscopic images

Clinical images						
Metric	Melanoma		Melanocytic naevus		Overall	
	ChatGPT-4o	Gemini 2.0 Flash	ChatGPT-4o	Gemini 2.0 Flash	ChatGPT-4o	Gemini 2.0 Flash
Response %	100%	95.6%	100%	97.1%	100%	95.6%
Correct diagnosis	90.6%	79.6%	49.2%	70.1%	64.4%	73.6%
Sensitivity	90.6% (85% to 94.2%)	79.6% (72.5% to 85.2%)	49.3% (43.4% to 55.1%)	68.1% (62.4% to 73.3%)	64.4% (59.8% to 68.7%)	72.2% (67.8% to 76.2%)
Specificity	79.4% (74.2% to 83.7%)	90.6% (86.6% to 93.5%)	96.9% (93.1% to 98.7%)	88.7% (82.8% to 92.7%)	85.9% (82.4% to 88.9%)	89.9% (86.7% to 92.4%)
Positive predictive value	71.6% (65.1% to 77.4%)	82.3% (75.4% to 87.6%)	96.5% (91.9% to 98.5%)	91.3% (86.6% to 94.4%)	81.9% (77.4% to 85.6%)	87.5% (83.7% to 90.6%)
Negative predictive value	93.6% (89.7% to 96.1%)	88.9% (84.8% to 92.1%)	53.2% (47.5% to 58.8%)	61.6% (55.1% to 67.6%)	70.9% (66.9% to 74.6%)	76.7% (72.8% to 80.1%)
Positive likelihood ratio	4.4	8.5	15.9	6	4.6	7.1
Negative likelihood ratio	0.12	0.23	0.52	0.36	0.41	0.31
Accuracy	83.5% (79.6% to 86.8%)	86.7% (83.1% to 89.8%)	67.1% (62.4% to 71.4%)	75.6% (71.3% to 79.6%)	75.2% (72.2% to 78%)	81.1% (78.3% to 83.7%)

Dermoscopic images						
Metric	Melanoma		Melanocytic naevus		Overall	
	ChatGPT-4o	Gemini 2.0 Flash	ChatGPT-4o	Gemini 2.0 Flash	ChatGPT-4o	Gemini 2.0 Flash
Dermoscopic image availability (%)	72.3%		93%		85.5%	
Response (%)	100%	87.8%	100%	97.3%	100%	94.4%
Correct (%)	96.5%	56.4%	27.6%	89.5%	48.9%	81.2%
Sensitivity	96.5% (91.4% to 98.6%)	56.4% (46.7% to 65.7%)	27.6% (22.5% to 33.4%)	91.2% (87% to 94.1%)	48.9% (43.9% to 54%)	81.2% (76.8% to 84.9%)
Specificity	53.7% (47.6% to 59.7%)	98.8% (96.5% to 99.7%)	100% (96.8% to 100%)	66.9% (57.9% to 74.9%)	68% (63.1% to 72.6%)	88.8% (85.1% to 91.6%)
Positive predictive value	48.3% (41.9% to 54.7%)	95% (86.3% to 98.6%)	100% (94.9% to 100%)	85.7% (81% to 89.4%)	60.5% (54.8% to 65.8%)	87.4% (83.4% to 90.6%)
Negative predictive value	97.2% (93% to 98.9%)	84.9% (80.3% to 88.5%)	38.2% (32.9% to 43.8%)	77.8% (68.6% to 84.8%)	57.1% (52.5% to 61.6%)	83.1% (79% to 86.5%)
Positive likelihood ratio	2.1	47	—	2.8	1.5	7.3
Negative likelihood ratio	0.07	0.44	0.72	0.13	0.75	0.21
Accuracy	66.9% (61.9% to 71.7%)	86.6% (82.6% to 90%)	50% (44.8% to 55.2%)	83.6% (79.4% to 87.2%)	58.5% (54.8% to 62%)	85.1% (82.2% to 87.6%)

Note: This table presents the diagnostic performance of ChatGPT-4o and Gemini 2.0 Flash in detecting melanoma and melanocytic naevi using clinical and dermoscopic images. The table includes key performance metrics: correct diagnosis rate, sensitivity, specificity, positive predictive value, negative predictive value, positive likelihood ratio, negative likelihood ratio and accuracy. Values are reported separately for melanoma, melanocytic naevi, and overall cases, with 95% confidence intervals (CIs) where applicable. The clinical and dermoscopic image performances are shown separately, with an additional row indicating the proportion of cases analyzed using dermoscopic images.

Phillips et al (2019) reported a specificity of 45.5% at 95% sensitivity using a digital single-lens reflex (DSLR) camera-based melanoma detection algorithm,^4^ while ChatGPT Vision (September 2023) had a sensitivity of 32%, specificity of 40%, and accuracy of 36%.^5^ Compared with earlier convolutional approaches, the models we tested use transformer-based vision encoders (in prior systems, often Vision-Transformer–style), though the exact backbones for the versions evaluated are not publicly disclosed. These encoders process images as grids of small “patches” and use attention to weigh information across the entire image, enabling consideration of whole-lesion patterns (eg, asymmetry, border irregularity, color variegation) rather than only local details. These systems are typically pretrained on large image collections with self-supervised objectives (eg, predicting masked regions), which can improve robustness. These characteristics may explain the higher sensitivity and/or specificity we observed relative to older DSLR-based algorithms. However, because we evaluated off-the-shelf models without fine-tuning and on a different dataset, performance differences cannot be attributed to architecture alone.

This study highlights the complementary strengths of ChatGPT-4o and Gemini 2.0 Flash, demonstrating that when both models were used, at least one identified melanoma in 96.2% of cases. This suggests that, with further training, such AI models could be integrated into dermatologic workflows, particularly for triaging high-risk lesions in settings with limited access to dermatologists or specialist care.

## Conflicts of interest

None disclosed.
